# Hilar en bloc resection for hilar cholangiocarcinoma in patients with limited liver capacities—preserving parts of liver segment 4

**DOI:** 10.1007/s10353-017-0507-8

**Published:** 2018-01-02

**Authors:** Sven Jonas, Felix Krenzien, Georgi Atanasov, Hans-Michael Hau, Matthias Gawlitza, Michael Moche, Georg Wiltberger, Johann Pratschke, Moritz Schmelzle

**Affiliations:** 1 Department of Surgery, 310Klinik, Nürnberg, Germany; 2 Department of Surgery, Campus Virchow-Klinikum and Campus Mitte, Charité—Universitätsmedizin Berlin, 13353 Berlin, Germany; 3 Department of Visceral, Transplant, Thoracic and Vascular Surgery, University Hospital Leipzig, Leipzig, Germany; 4 Department of Diagnostic and Interventional Radiology, 310Klinik, Nürnberg, Germany

**Keywords:** Klatskin tumor, Cholangiocarcinoma, Margins of excision, Hepatectomy, Liver diseases

## Abstract

**Background:**

A right trisectionectomy with portal vein resection represents the conventional approach for hilar cholangiocarcinoma. Here, we present a technical modification of hilar en bloc resection in order to increase the remnant volume by partially preserving liver segment 4.

**Methods:**

The caudal parenchymal dissection line starts centrally between the left lateral and left medial segments. Cranially, the resection line switches to the right towards Cantlie’s line and turns again upwards perpendicularly. Hence, segment 4a and subtotal segment 4b are partially preserved by this novel technique. The left hepatic duct is dissected at the segmental ramification and reconstruction is performed as a single hepaticojejunostomy. The feasibility of the novel parenchyma-sparing approach for hilar cholangiocarcinoma was proven in a case series and medical records were reviewed retrospectively.

**Results:**

Ten patients (6 male, 4 female) underwent segment 4 partially preserving right trisectionectomy for hilar cholangiocarcinoma. Estimated future liver remnant volume was significantly increased (FLRV 38.3%), when compared to standard right trisectionectomy (FLRV 23.9%; *p* < 0.01). Three of 10 liver resections were associated with major surgical complications (≥IIIb; *n* = 3); categorized according to the Dindo–Clavien classification. No patient died due to complications associated with postoperatively impaired liver function. Tumor-free margins could be achieved in 8 patients while median overall survival and disease-free survival were 547 and 367 days, respectively.

**Conclusion:**

This novel parenchyma-sparing modification of hilar en bloc resection by partially preserving segment 4 allows to safely increase the remnant liver volume without neglecting principles of local radicality.

**Electronic supplementary material:**

The online version of this article (10.1007/s10353-017-0507-8) contains supplementary material, which is available to authorized users.

Here, we report on a novel, parenchyma-sparing modification of right trisectionectomy with hilar en bloc resection for hilar cholangiocarcinoma. The surgical approach allows to partially preserve liver segment 4 to increase the remnant liver volume without neglecting principles of radicality.

## Introduction

In the treatment of hilar cholangiocarcinoma, experienced centers have proceeded to perform extended liver resections instead of extrahepatic bile duct resections as early as in the 1980s [1–5]. Liver resection was, and in most centers still is, extended to the side of the liver of predominant tumor growth. Using this approach, 5‑year survival rates increased to around 20 to 40%, which was a significant step forward in comparison to extrahepatic bile duct resections that did not result in significant survival in the long-term [6, 7].

After we had conceptualized the oncological advantage of a combined right trisectionectomy and hilar en bloc resection, the Berlin concept was, henceforth, performed prospectively and has recently been analyzed [8]. A 5-year survival rate of 58% after R0 resection could be achieved in spite of extending indications even to some hilar cholangiocarcinomas extending to the left. A limitation of right trisectionectomy, irrespective of adding or not adding portal vein resection, is a frequently small left-lateral remnant liver section in spite of preoperative portal vein embolization (PVE). In this recent analysis, morbidity related to liver failure was 30% and significantly exceeded the rate of 16% in all other types of conventional liver resection for hilar cholangiocarcinoma. Indeed, liver function confines patient suitability for extended liver resections.

Herein, we report for the first time on a novel parenchyma-sparing modification of hilar en bloc resection for hilar cholangiocarcinoma. The surgical approach allows to partially preserve liver segment 4 in order to increase the remnant liver volume and to facilitate surgical radicality.

## Methods

### Patient characteristics

We reviewed the medical records of 10 patients who underwent segment 4 partially preserving extended right hepatectomy for hilar cholangiocarcinoma. Hilar cholangiocarcinoma was verified histopathologically and classified according to the Bismuth–Corlette and Union International contre le cancer TNM classification, respectively. Patient data were analyzed with regard to the feasibility and safety of this novel surgical technique. The analysis was approved by the local ethic committee (AZ 243-14-14072014). Please see electronic Supplementary Methods for further information.

### Technical intraoperative aspects

We here present a modification of right trisectionectomy with portal vein resection, based on the NEUHAUS procedure of our Berlin concept (1999), where segment 4 is partially preserved. The main focus of this modification was to increase the remnant liver volume to the highest possible amount and to provide an oncologically safe approach at the same time. In detail, the liver was first mobilized from its ligaments and the inferior vena cava. Lymphadenectomy was performed along the left margin of the hepatoduodenal ligament to the superior margin of the pancreas, down the common hepatic artery to the celiac trunk. The tumor-bearing area has been spared from the lymphadenectomy in order to perform an en bloc resection and to comply with the no-touch concept. These steps were followed by preparation and isolation of the right hepatic vein, and dissection of the right hepatic artery at its origin. The left hepatic artery was isolated along its entire course (Fig. 1a). The left portal branch was isolated and small branches to segments 1 and 4 were dissected. Division of the bile duct was performed distal to the cystic duct at the superior margin of the pancreas. The portal vein trunk was isolated and closed with a vascular clamp. Resection of the portal vein bifurcation was performed. Immediately after resection, end-to-end anastomosis of the portal trunk to the left portal vein branch was conducted (Prolene 6.0) with the need for postoperative systemic heparin for anticoagulation (Fig. 1b and c).

**Fig. 1:**
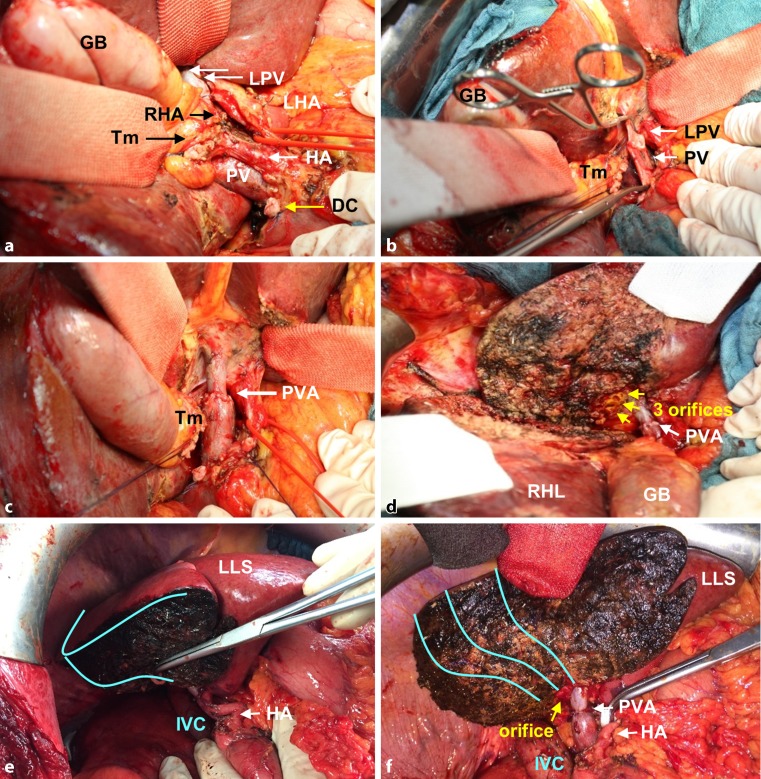
Intraoperative situation during a segment 4 partially preserving hilar en bloc resection with principle portal vein resection for the treatment of hilar cholangiocarcinoma. **a** After completion of a lymphadenectomy down to the celiac trunk, the choledochal duct (*DC*; *yellow arrow*) has been divided in projection to the superior margin of the pancreatic head. The tumor bearing area (*Tm*) has been spared from the lymphadenectomy in order to allow a no-touch resection and to comply with the principle of a no-touch technique. The right hepatic artery (*RHA*; only the ligated stump is visible) has been divided at its junction with the proper hepatic artery (*AH*) and crosses behind the tumor. In contrast, the left hepatic artery (encircled by a red rubber loop) runs in distance to the tumor and can easily be isolated up to the umbilical fissure. The main trunk of the portal vein (*PV*) and the left portal vein branch (*LPV*) have already been prepared for clamping and division later on (*GB* gallbladder). **b** The main trunk of the portal vein (*PV*) and the left portal vein branch (*LPV*) have been occluded with vascular clamps superior to the duodenal bulb and within the umbilical fissure, respectively. On the side of the tumor bearing area (*Tm*), occlusion will be performed with ligations. **c** Division and reconstruction with an end-to-end anastomosis (*PVA*; *white arrow*) between the portal vein trunk and the left portal vein branch. After reconstruction, the slightly stretched course of the portal vein gives a harmonic appearance and avoids a typical kinking which sometimes results after extended right hepatic resection combined with caudate lobectomy. **d** Division of the left hepatic duct in a macroscopically tumor-free portion, i. e., generally just before or beyond the segmental ramification, resulting in three orifices (*yellow arrows*) draining segments 2, 3, and 4 (*RHL* right hemiliver, *GB* gallbladder, *PVA* portal vein anastomosis). **e,f,h** Parenchymal transection: The anterior parenchymal resection line started in Cantlie’s line and remained midline in the cranial portion of the liver throughout the transection. In the caudal portion, the resection line turned to the left (*light blue lines*), surrounding the perihilar region at a distance of 2–3 cm until it reached the border between the left lateral section (*LLS*) and segment 4 (*S4*) where it again ran perpendicularly (*IVC* inferior vena cava)

**Fig. 1 (continued):**
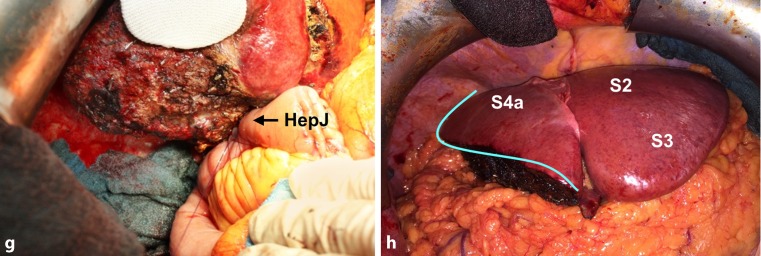
Intraoperative situation during a segment 4 partially preserving hilar en bloc resection with principle portal vein resection for the treatment of hilar cholangiocarcinoma. **e, f, h** Parenchymal transection: The anterior parenchymal resection line started in Cantlie’s line and remained midline in the cranial portion of the liver throughout the transection. In the caudal portion, the resection line turned to the left (*light blue lines*), surrounding the perihilar region at a distance of 2–3 cm until it reached the border between the left lateral section (*LLS*) and segment 4 (*S4*) where it again ran perpendicularly (*IVC* inferior vena cava) **g** Reconstruction as a single hepaticojejunostomy (*HepJ*) after partial reunification of the ducts with single stiches (polydioxanone 5.0 or 6.0)

The anterior parenchymal resection line started in Cantlie’s line (the line runs between segment 4 on the left and segments 5 and 8 on the right) and remained midline in the cranial portion of the liver throughout the transection. In the caudal portion, the resection line turned to the left surrounding the perihilar region at a distance of 2–3 cm until it reached the border between the left lateral (segment 3) and left medial (segment 4b) section (Fig. 1d–h). The parenchymal dissection was finalized in this intersection plane running perpendicularly, which corresponds to the Berlin concept by using a no-touch technique and the full length of the left hepatic duct to gain as wide a tumor-free margin as possible. This technique is, of course, very much in contrast to former approaches, which included a lowering of the hilar plate. A caudate lobectomy was always included. Hence, major parts of segment 4a and subtotal segment 4b are preserved by this technique. Ideally, the central venous pressure was held below 4 mm Hg during parenchymal transection. Finally, the left hepatic duct was divided in a macroscopically tumor-free portion, i. e., generally just before or beyond the segmental ramification, resulting in one to three ostia draining segments 2, 3, and 4 (Fig. 1d). Tumor-free margins were confirmed by frozen section examination. Reconstruction was performed as a single hepaticojejunostomy after partial reunification of the ducts with single stiches (polydioxanone, PDS, 5.0 or 6.0) and using a preoperatively placed percutaneous transhepatic cholangiography and drainage (PTCD) or intraoperatively placed transhepatic NEUHAUS drainage for decompression and postoperative cholangiography. Postoperative cholangiography was routinely performed after 1 week prior to closure of the drainage.

## Results

### General characteristics and follow-up

Ten patients (6 male, 4 female) with a median age of 70 years (range 38–79 years) undergoing segment 4 partially preserving right trisectionectomy for hilar cholangiocarcinoma were analyzed with regard to perioperative safety and oncological radicality (Supplementary Results). Median body mass index was 24 (range 20–30). Median follow up after liver resection was 612 days (range 367–1983 days). Median overall survival and disease-free survival were 547 days (range 9–1983 days) and 367 days (range 9–886 days), respectively (Supplementary Fig. 1). One patient died within 90 days (postoperative day 9) due to acute pulmonary emboli, without evidence for a postoperative deterioration of the liver function. Three patients developed recurrent disease 12 months (2 patients) and 29 months after operation. Two of these patients underwent formally curative surgery for recurrent tumor and are still alive, whereas the latter patient died during follow-up. Two patients died due to physical impairment without evident recurrent disease 5 and 7 months after the operation.

### Surgical characteristics

**Fig. 2:**
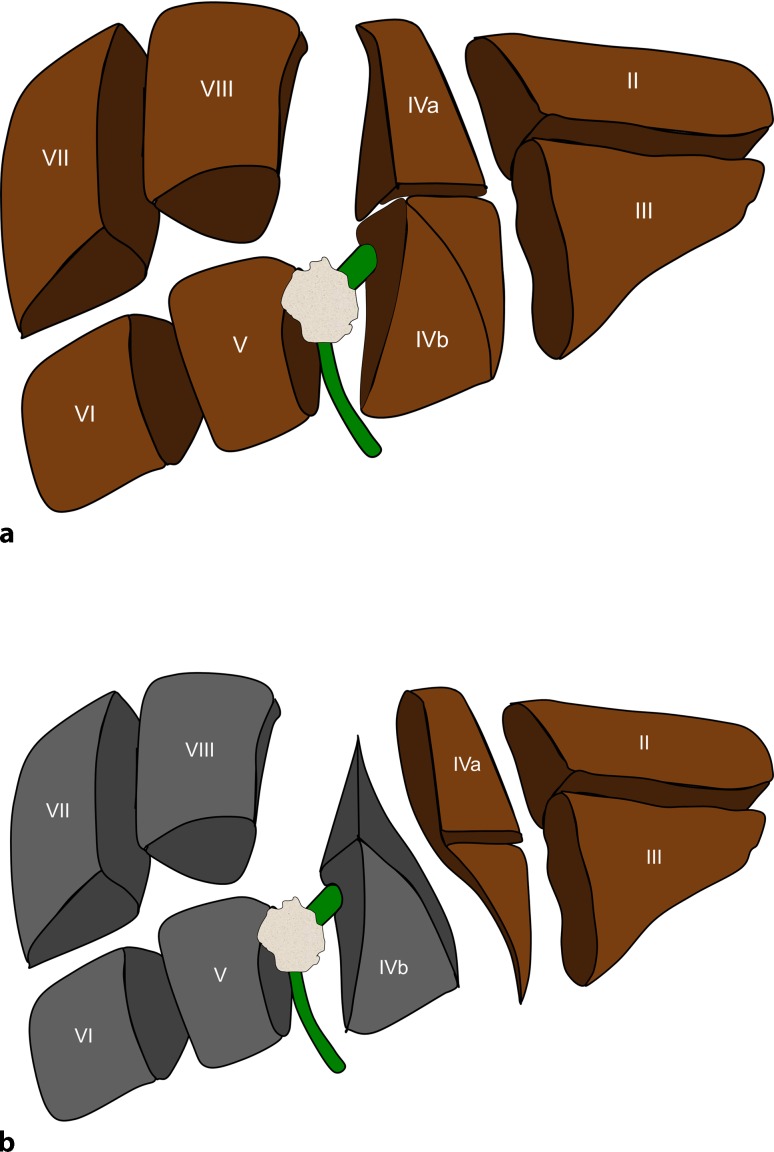
Intraoperative situation during a segment 4 partially preserving hilar en bloc resection with a focus on principles of the resection line, the division of the left hepatic duct, and the reconstruction by hepaticojejunostomy. **a,b** Illustration of the resection line after parenchymal transection: the cranial parenchymal resection starts in Cantlie’s line before it turns to the left, perpendicularly between the left medial and the left lateral section, resulting in a partial resection of segment 4

**Fig. 3:**
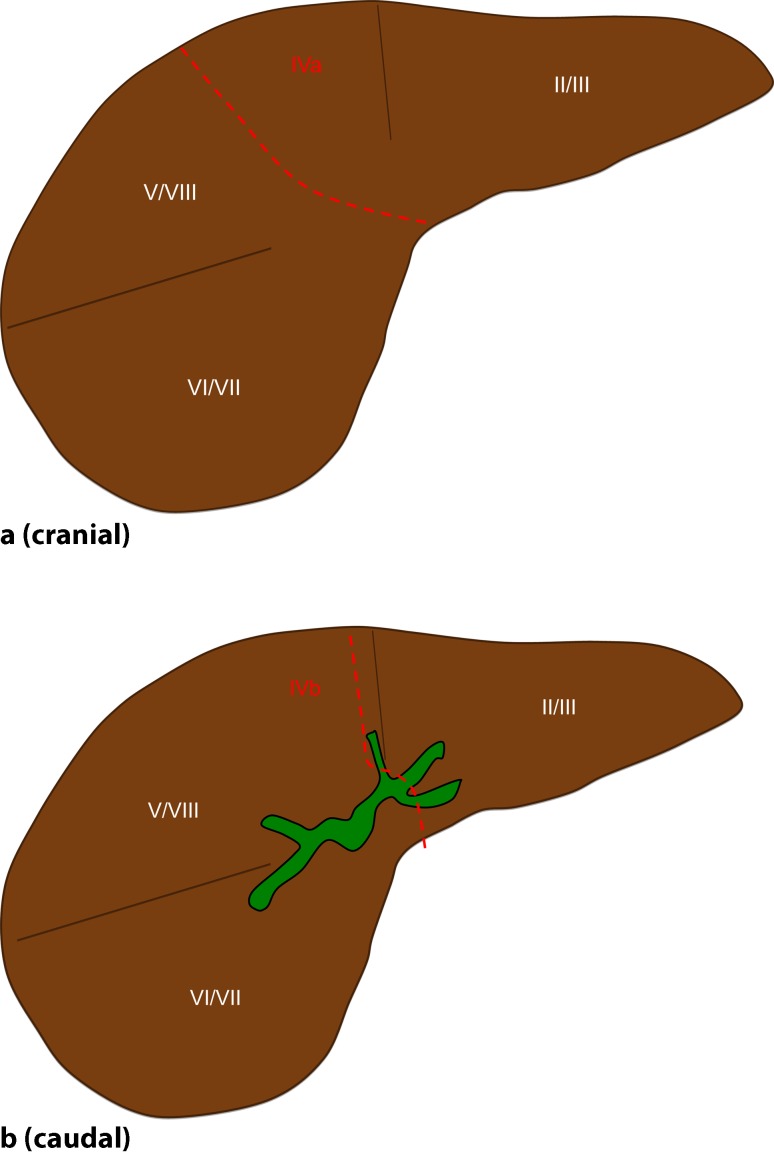
Intraoperative situation during a segment 4 partially preserving hilar en bloc resection with f shape of the transection line. **a** represents the transversial section through the cranial part of the liver**, b** represents the transversial section through the caudal part of the liver

**Fig. 4:**
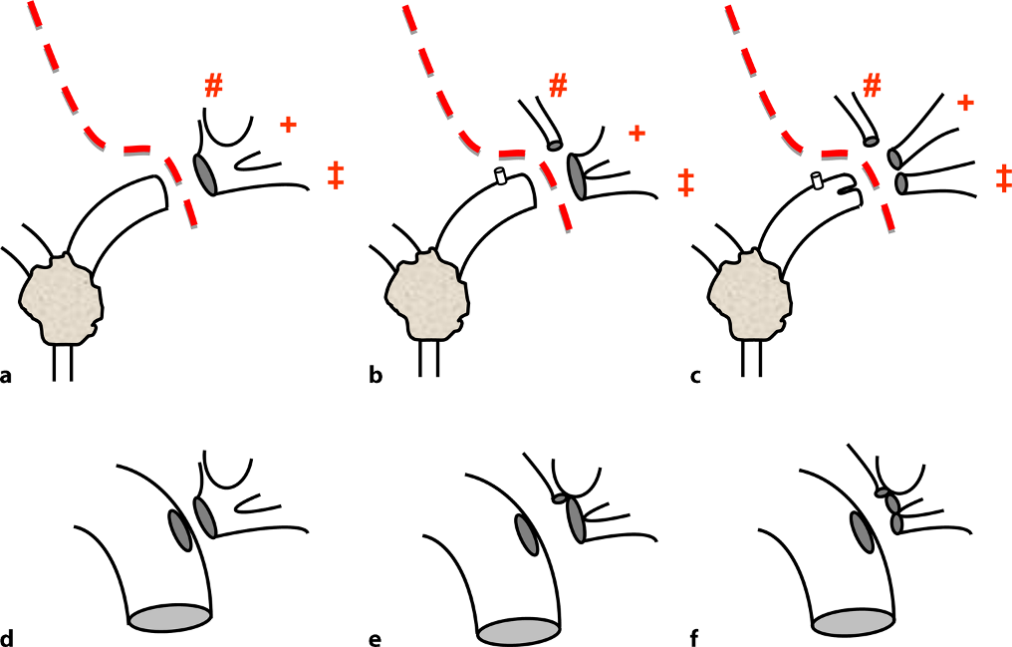
Principles of division of the left hepatic duct and reconstruction as a single hepaticojejunostomy. Division of the left hepatic duct in a macroscopically tumor-free portion, just before (**a**) or beyond (**b,c**) the segmental ramification, resulting in one (**a**), two (**b**), or three (**c**) orifices draining segments 2 (*‡*), 3 (*+*), and 4 (*#*). Reconstruction of the left bile duct (**d**) or after partial reunification of the ducts draining segments 2, 3 and 4 (**e,f**) as a single hepaticojejunostomy, as needed. Intraluminal decompression and splinting using a transhepatic NEUHAUS drainage not shown for didactical reasons

Hepatic arterial vascular anatomy was classified according to Michels’ classification, with normal anatomy (type 1) in 7 patients, left hepatic artery from left gastric artery (type 5) in 1 patient, and right hepatic artery from superior mesenteric artery (type 6) in 2 patients. Following parenchymal transection (Figs. 2 and 3), one bile duct ostium (distal left hepatic duct) resulted in 3 patients, two bile duct ostia (one ostium draining segments 2 and 3; one ostium draining segment 4a) in 4 patients, and three ostia (segmental ostia draining segments 2, 3, and 4a, respectively) in 3 patients (Fig. 4). The resulting two or more bile duct ostia were reunified by suture, so placing of a NEUHAUS drain to one single hepatojejunostomy (PDS 5/0 or 6/0) was technically feasible in all cases. We routinely place NEUHAUS drains for postoperative decompression and splinting of the tiny anastomosis, unless an existing PTCD can be used instead. Bile duct stents were removed intraoperatively. Bile duct decompression was routinely extended to the postoperative course by using a preoperatively placed PTCD in 2 patients or an intraoperatively placed transhepatic NEUHAUS drain in 6 patients. In 2 patients, two separate transhepatic NEUHAUS drains were used (to segments 3 and 4a).

### Liver function

According to the preoperative CT volumetry, median TLV was 1706.3 ml (range 1413.8–2568.3 ml). The %FLRV for segments 2 and 3 was 23.9% (range 15.1–39.2%), and significantly lower when compared to the %FLRV for segments 2–4 of 38.3% (range 24.1–58.1%; *p* = 0.007). A total of 5 patients suffered from a %FLRV below 25% (median 16.8%; range 15.1–23.9%) for segments 2–3, which is considered necessary for a functionally safe liver resection. The segment 4 partially preserving approach allowed all patients to safely undergo extended right liver resection. In 5 patients (50%), preoperative PVE was performed additionally to the planned segment 4-preserving approach for additional liver augmentation of the left lateral segments. The reasons for partially preserving segment 4 in patients with a %FLRV >25% were signs of a restricted cholestatic liver damage, steatosis, or fibrosis.

In patients with postoperative albumin levels <30 g/dl, a trend was observed towards an increase in serious complications with the need for intervention or re-operation and a prolonged hospital stay (median: 60 postoperative days vs. 37 postoperative days; *p* = 0.18).

### Clinical course and surgical complications

Median total length of stay on the intensive care unit was 5 days (range 1–19 days) and median length of hospital stay was 40 days (range 10–148 days). Nine of 10 liver resections were associated with complications in the postoperative course, categorized according to the Dindo–Clavien classification as IIIa (*n* = 4, 40%), IIIb (*n* = 3, 30%), IV (*n* = 1, 10%), and V (*n* = 1, 10%). In 4 of 10 patients (40%), abdominal re-exploration was required due to intraabdominal hematoma (*n* = 1), suspected abdominal bleeding due to lysis of acute pulmonary emboli (*n* = 1), fascial dehiscence (*n* = 1), and insufficiency of hepaticojejunostomy (*n* = 1).

### Histopathological characteristics

Hilar cholangiocarcinoma was verified histopathologically in all cases, with a median tumor size of 2.5 cm (range 0–4.5 cm). Tumors were classified according to the Bismuth–Corlette classification as type I (*n* = 1; 10%), type III a (*n* = 1; 10%), type III b (*n* = 1; 10%), and type IV (*n* = 7; 70%), and according to the TNM classification as stage II (*n* = 7; 70%), III (*n* = 2; 20%), and IV (*n* = 1; 10%). Histopathological differentiation was moderate (G2) in 3 patients and poor (G3) in 7 patients. Positive lymph nodes (2/11) were detected in 1 patient and a distant metastasis in liver segment 5 was evident in 1 patient.

Microscopically tumor-free resection margins (R0) could be achieved in 8 patients (80%). In 1 patient each suffering from a Bismuth type IV cholangiocarcinoma, microscopic residual disease (R1 resection) was diagnosed postoperatively in the left hepatic periductal tissue and in a third-order branch draining segment 3 (please see electronic Supplementary Results for further information).

## Discussion

This is the first report of a conceptualized segment 4 partially preserving modification of right trisectionectomy combined with a hilar en bloc resection for the treatment of hilar cholangiocarcinoma. This novel modification of the Berlin concept reconciles the oncological advantages of our conventional approach, i. e., right trisectionectomy and portal vein resection, with an increase in safety by preserving parts of the liver parenchyma of segment 4. No patient died from the reportedly most common cause of postoperative mortality, i. e., postoperative acute liver failure (POLF), which justifies the rationale for preserving segment 4. This demise of mortality associated to liver failure is in contrast to our previous reports on right trisectionectomy, with hilar en bloc resection and mortality rates of 12% [8].

Partially preserving segment 4 is likely to have resulted in exclusion of some patients who, due to their tumors’ growth pattern, rather required a right trisectionectomy. However, most patients (7 of 10 patients) in the present series suffered from Bismuth type IV tumors, with intrahepatic tumor spread to liver segment 5 in 1 patient. R0 resections were achieved in 8 of 10 patients at the site of the hepatic and biliary resection plane, including 5 of 7 patients suffering from Bismuth type IV tumors. Currently, 3 of 7 patients suffering from Bismuth type IV tumors are alive without recurrence 1, 2, and 3 years after resection. Therefore, Bismuth type IV hilar cholangiocarcinomas are not a contraindication per se to a segment 4 partially preserving right trisectionectomy with hilar en bloc resection; tumor-free margins may still be achieved if the tumor does not extend too far into the left hepatic third-order ducts. This is important to note, because Bismuth type IV tumors had been considered unresectable until recently [9, 10]—with that opinion being included in the American Joint Committee on Cancer (AJCC) staging manual (7th edition) [11]. Han et al. from Korea have presented an elaborate surgical series of 33 patients with a 64% R0 resection rate and a 3-year survival rate of 28%. They concluded that, with careful selection, patients suffering from Bismuth type IV tumors may expect prolonged survival and should be considered as candidates for resection [12].

Leakage from a hepaticojejunal anastomosis occurred in 1 patient. Generally, treatment will be prolonged and sometimes cumbersome. The mainstay of the treatment strategy is drainage of the intraabdominal collection and biliary decompression via the NEUHAUS drain which is already in place. In addition, rinsing the site of leakage and collection may facilitate the formation of adhesions by diluting bile and debris. Moreover, we experienced the exchange of a PTCD for a NEUHAUS- drain as a decisive step towards increased drainage volumes and desiccation of the biliary fistula. Prophylactic measures include a meticulous anastomotic technique using monofilament single-stitch sutures (PDS 5.0) and magnification glasses.

In addition, hypoalbuminemia should ideally be avoided in order to keep the colloid-osmotic pressure in a normal range, thus protecting the intestine from edema and, in consequence, the anastomosis from strain as well as the threads in the tiny biliary wall from traction. In the present study, albumin levels significantly decreased during the postoperative course. Despite the small number of patients, hypoalbuminemia below 30 g/dl tended to correlate with the need for reintervention or reoperation. However, we cannot conclusively distinguish between cause and effect of hypoalbuminemia and complications, it may be hypothesized that albumin serum levels were inadequately corrected [13]. In a meta-analysis of nine randomized trials on exogenous albumin administration in a variety of acute diseases, attaining a serum albumin level of more than 30 g/dl was shown to significantly decrease complication rates [14], in line with the more recent SAFE trial [15].

The crucial question remains of which patient should be offered which procedure. If tumor extent and tumor growth are not an obstacle, then the ideal approach, in our view, is the Berlin concept of Neuhaus et al., i. e., right trisectionectomy with hilar en bloc resection including resection of the portal vein bifurcation [6, 7]. If patients are considered ineligible due to inadequate remnant volume or parenchymal quality of segments 2 and 3, right portal vein embolization and selective left-sided biliary decompression should be performed—if not done already. Those patients who, due to liver volume or quality, are still not eligible to undergo a right trisectionectomy are, in our hands, candidates for a segment 4 partially preserving extended right hepatectomy with hilar en bloc resection, as presented in this study. In some patients, left hepatic resections may be possible. However, principles of en bloc resection and the no-touch technique can hardly be complied with by left-sided liver resections [16]. In addition, Bismuth type IV tumors are highly unlikely to be amenable to R0 resections by left hepatic resections, due to the extremely short distance between the hepatic bifurcation and second-order ramifications.

Tactics to increase and accelerate liver hypertrophy as well as to enable patients to undergo right trisectionectomy in spite of a limited left-lateral remnant section include the novel surgical concept of ALPPS (associating liver partition and right portal vein ligation for staged hepatectomy). The ALPPS concept includes a two-step operation, without waiting time for the first step and induction of rapid liver hypertrophy within the very short period of 7–10 days until the second step [17]. In the first step, ligation of the right portal vein branch and parenchymal transection are performed, followed by completion hepatectomy of the right side in the second step. Unfortunately, an analysis of the largest international registry (NCT01924741) has recently confirmed earlier single-institution reports on increased morbidity and mortality rates when performing ALPPS in patients suffering from hilar cholangiocarcinoma [18–20]. The registry data disclosed that postoperative 90-day mortality was highest in patients suffering from hilar cholangiocarcinoma or gallbladder cancer, reaching 27% and 33%, respectively. It was concluded that ALPPS should be performed with great caution in this population, if at all.

Results from non-randomized clinical treatment attempts evaluating the regenerative potential of preoperative left intraportal administration of autologous hematopoietic stem cells in addition to a right portal vein embolization are promising and confirmed by various experimental studies in rodents [21, 22].

## Conclusion

We here report a conceptualized segment 4-preserving approach of an extended right hepatectomy combined with hilar en bloc resection for the treatment of hilar cholangiocarcinoma. This novel technical modification reconciles the oncological advantages of our conventional approach with an increase in safety by preserving most of the liver parenchyma of segment 4. Patients considered ineligible for right trisectionectomy with hilar en bloc resection due to inadequate remnant volume or parenchymal quality of segments 2 and 3 are preferred candidates for the segment 4-preserving approach. Future studies need to clarify whether this modification procedure might even be accepted as the new standard in patients with hilar cholangiocarcinoma.

## Caption Electronic Supplementary Material

Methods and results

Supplementary Fig. 1 Kaplan–Meier survival curve representing the follow-up and cumulative survival probability after segment 4 partially preserving hilar en bloc resection for hilar cholangiocarcinoma

## Abbrevations

PVE: Portal vein embolization
MRCP: Magnetic resonance cholangiopancreatography
ERC: Endoscopic retrograde cholangiography
PTCD: Percutaneous transhepatic cholangiography drainage .
TLV: Total liver volume
FLRV: Future liver remnant volume
ALT: Alanine aminotransferase
AP: Alkaline phosphatase
GGT: Gamma-glutamyl transpeptidase
ALPPS: Associating liver partition and right portal vein ligation for staged hepatectomy
