# Correction to: Hilar en bloc resection for hilar cholangiocarcinoma in patients with limited liver capacities—preserving parts of liver segment 4

**DOI:** 10.1007/s10353-018-0559-4

**Published:** 2018-11-05

**Authors:** Sven Jonas, Felix Krenzien, Georgi Atanasov, Hans-Michael Hau, Matthias Gawlitza, Michael Moche, Georg Wiltberger, Johann Pratschke, Moritz Schmelzle

**Affiliations:** 1 Department of Surgery, 310Klinik, Nürnberg, Germany; 2 Department of Surgery, Campus Virchow-Klinikum and CampusMitte, Charité—Universitätsmedizin Berlin, 13353 Berlin, Germany; 3 Department of Visceral, Transplant, Thoracic and Vascular Surgery, University Hospital Leipzig, Leipzig, Germany; 4 Department of Diagnostic and Interventional Radiology, 310Klinik, Nürnberg, Germany

**Correction to:**

**Eur Surg 2017**

10.1007/s10353-017-0507-8

The article “Hilar en bloc resection for hilar cholangiocarcinoma in patients with limited liver capacities—preserving parts of liver segment 4” written by Sven Jonas, Felix Krenzien, Georgi Atanasov, Hans-Michael Hau, Matthias Gawlitza, Michael Moche, Georg Wiltberger, Johann Pratschke, and Moritz Schmelzle was originally published electronically on the publisher’s Internet portal (currently SpringerLink) on 2 January 2018 without open access.

With the author(s)’ decision to opt for Open Choice, the copyright of the article changed in November 2018 to © The Author(s) 2018 and the article is forthwith distributed under the terms of the Creative Commons Attribution 4.0 International License (http://creativecommons.org/licenses/by/4.0/), which permits use, duplication, adaptation, distribution, and reproduction in any medium or format, as long as you give appropriate credit to the original author(s) and the source, provide a link to the Creative Commons license, and indicate if changes were made.

