# Supplementary material for: Hilar en bloc resection for hilar cholangiocarcinoma in patients with limited liver capacities—preserving parts of liver segment 4
Source: Eur Surg. 2018 Jan 2;50(1):22–9. doi: 10.1007/s10353-017-0507-8 (PMC6223732; doi:10.1007/s10353-017-0507-8)
Supplement: Supplementary file 1 — Methods and results [file 10353_2017_507_MOESM1_ESM.doc]

**Supplementary Methods**

Patients were analyzed for perioperative safety und oncological radicality of the technical modification proposed in the present study. If this approach was considered technically feasible without multiorgan resection and functionally safe (i.e. preoperative serum albumin levels > 30mg/dl and a FLRV of segments 2 - 4 of not less than ≈ 25% of TLV). Neither suspected metastasis to regional lymph nodes nor tumor infiltration in the portal vein were considered as general contraindications to curative surgery. Surgery was not considered suitable for patients with distant metastases or peritoneal carcinomatosis.

*Preoperative management*

Patients underwent routine staging using preoperative helical computed tomography scanning of the abdomen and pelvis. In case of anatomic variations or if tumor characteristics were not clearly definable on computed tomography scans, magnetic resonance imaging with cholangiopancreatography (MRCP) was additionally performed. Hepatic duct decompression was considered in those patients who did not already present with a stent on admission and/or in whom the serum bilirubin level exceeded 8 mg/dl; it was achieved via endoscopic retrograde cholangiography (ERC) and stent and/or percutaneous transhepatic cholangiography drainage (PTCD). Total liver volume (TLV) and the future liver remnant volume (FLRV) of left lateral liver segments 2 and 3 and the left medial segment 4, respectively, were estimated preoperatively by computed tomography-volumetry. Preoperative PVE for liver augmentation was performed in selected patients.

*Variables / data collection*

Complications were assessed according to the Dindo-Clavien classification. The following variables were assessed: sex (male vs. female), age (years), preoperative PVE (yes vs. no), preoperative decompression via PTCD or stent (yes vs. no), duration of the operation (min), hilar occlusion (yes vs. no), portal vein reconstruction (yes vs. no), resulting bile duct ostia (n), resected liver volume (g), liver quality (good, < 20% fat, no siderosis, no fibrosis vs. “with restrictions”, > 20% fat, siderosis, fibrosis), tumor size (cm), resection margins (R0 vs. R1), left hepatic resection margin (R0 vs. R1), intraoperative red blood cell concentrate (yes vs. no), intraoperative fresh frozen plasma (yes vs. no), length of intensive care unit (intensive care unit) stay (days), length of hospital stay (days), overall survival (yes vs. no), overall survival (days), disease-free survival (days), 30- and 90-day mortality (yes vs no). Serum levels of albumin (g/l), prothrombin time (%), alanine aminotransferase (ALT, µkat/l), alkaline phosphatatse (AP, µkat/l), gamma-glutamyl transpeptidase (GGT, µkat/l), total bilirubin (µmol/l), and creatinine (µmol/l), were assessed preoperatively and on postoperative day 7, respectively.

*Statistics*

Data are presented as median (range) unless otherwise specified. Statistical differences between groups were determined by Student´s t - test. Survival data were analyzed using the log rank test.

**Supplementary Results**

*Preoperative management*

Staging computed tomography was performed in all patients, with additional cholangiography and MRCP performed in 9 and 7 patients, respectively. Preoperative decompression via PTCD or ERC and stent was considered advisable in 9 of 10 patients (90%) due to tumor-associated cholestasis. Cholestasis was treated in 7 patients by ERC and stent, with selective decompression of the left liver lobe in 4 patients and non-selective stenting of both left and right bile ducts in 3 patients. Preoperative PTCD was performed in 3 patients, with decompression of the left liver lobe in 2 patients and decompression of both liver lobes in 1 patient. In 1 of these patients, PTCD was required additionally to ERC and stent.

According to the preoperative computed tomography-volumetry median TLV was 1706.3 ml (range 1413.8 – 2568.3). The %FLRV for segments 2 and 3 was 23.9% (range 15.1 – 39.2) and significantly lower, when compared to the %FLRV for segments 2 – 4 of 38.3% (range 24.1 – 58.1) (p = 0.007). A total of 5 patients suffered from a %FLRV below 25% (median 16.8%; range 15.1 – 23.9) for segments 2 – 3, which is considered necessary for a functionally safe liver resection. The segment 4 partially preserving approach allowed all patients to safely undergo extended right liver resection. In 5 patients (50%), preoperative PVE was performed additionally to the planned segment 4 preserving approach for additional liver augmentation of the left-lateral segments. The reasons for partially preserving segment 4 in patients with a %FLRV > 25% were signs of a restricted cholestatic liver damage, steatosis, or fibrosis.

*Histopathological characteristics*

Median weight of the resected liver was 979 g (range 491 – 1464) with the liver quality being assessed postoperatively and considered “good” in 2 patients (20 %) and “with restrictions” in 8 patients (80 %).

Hilar cholangiocarcinoma was verified histopathologically in all cases, with a median tumor size of 2.5 cm (range 0 – 4.5). Tumors were classified according to the Bismuth-Corlette classification as type I (n = 1; 10%), type III a (n = 1; 10%), type III b (n = 1; 10%), and type IV (n = 7; 70%) and according to the TNM-classification as stage II (n = 7; 70%), III (n = 2; 20%) and IV (n = 1; 10%). Histopathological differentiation was moderate (G2) in 3 patients and poor (G3) in 7 patients. Positive lymph nodes (2/11) were detected in 1 patient and a distant metastasis in liver segment 5 was evident in 1 patient.

Microscopically tumor-free resection margins (R0) could be achieved in 8 patients (80%). In one patient each suffering from a Bismuth type IV cholangiocarcinoma, microscopic residual disease (R1-resection) was diagnosed postoperatively in the left hepatic periductal tissue and in a third order branch draining segment 3, respectively.

***Surgical characteristics***

Median duration of the operation was 529 min (range 355 - 553), with temporary hilar occlusion being necessary in 1 patient. Portal vein resection with immediate reconstruction represents our standard approach and was performed in 7 of 10 patients. A no touch technique approach, however, was possible in 3 patients without resection of the portal vein bifurcation due to a straight course of the left portal vein branch from the trunk and fluffy tissue surroundings void of any sign of adherence or even infiltration. In 3 patients, 3 and 2 red blood cell concentrates as well as 2 fresh frozen plasma were transfused patients intraoperatively, respectively.

***Liver function***

Median weight of the resected liver was 979 g (range 491 – 1464) with the liver quality being assessed postoperatively and considered “good” in 2 patients (20 %) and “with restrictions” in 8 patients (80 %).

In comparison to preoperative levels, serum albumin (41.4 vs. 26.8; p < 0.001) and prothrombin time (98% vs. 71%; p < 0.001) significantly decreased until postoperative day 7, whereas no significant changes were noted for serum levels of ALT (1.02 vs. 1.07; p = 0.32), AP (4.88 vs. 2.16; p = 0.1), GGT (6.51 vs. 3.64; p = 0.21), total bilirubin (18.0 vs. 34.7; p = 0.13), creatinine (71.5 vs. 73.5; p = 0.79), respectively.

***Complications and postoperative liver function***

Median total length of stay on intensive care unit was 5 days (range 1 - 19) and median length of hospital stay was 40 (range 10 - 148). Nine of 10 liver resections were associated with complications in the postoperative course, categorized according to the Dindo-Clavien classification as IIIa (n = 4, 40%), IIIb (n = 3, 30%), IV (n = 1, 10%) and V (n = 1, 10%). In 4 of 10 patients (40%), abdominal re-exploration was required due to intraabdominal hematoma (n = 1), suspected abdominal bleeding due to lysis of acute pulmonary emboli (n = 1), fascial dehiscence (n = 1) and insufficiency of hepaticojejunostomy (n = 1).

Insufficiency of the hepaticojejunostomy was noted in 1 patient (see above). However, leakage due to a small perforation of the jejunal loop 1 cm beside the hepaticojejunostomy was evident in another patient. This patient was treated percutaneously interventionally and discharged with a drainage. Interventional drainage of perihepatic hematoma/abscesses was performed in 2 patients. Postoperative bleeding complications were evident in 2 patients, with diffuse intraabdominal bleeding after systemic lysis due to acute pulmonary emboli (n = 1) as mentioned above and bleeding of the middle hepatic vein (n = 1), which was stopped by angiography and interventional stenting. No patient died due to complications associated to impaired liver function.

In comparison to preoperative levels, serum albumin (41.4 vs. 26.8; p < 0.001) and prothrombin time (98% vs. 71%; p < 0.001) significantly decreased until postoperative day 7, whereas no significant changes were noted for serum levels of ALT (1.02 vs. 1.07; p = 0.32), AP (4.88 vs. 2.16; p = 0.1), GGT (6.51 vs. 3.64; p = 0.21), total bilirubin (18.0 vs. 34.7; p = 0.13), creatinine (71.5 vs. 73.5; p = 0.79), respectively. No patient died due to complications associated to impaired liver function.

In patients with postoperative albumin levels < 30 g/dl, a trend was observed towards an increase in serious complications with the need for intervention or re-operation and a prolonged hospital stay (median: 60 postoperative days vs. 37 postoperative days; p = 0.18).
