# Supplementary figures and images for: Hilar en bloc resection for hilar cholangiocarcinoma in patients with limited liver capacities—preserving parts of liver segment 4
Source: Eur Surg. 2018 Jan 2;50(1):22–9. doi: 10.1007/s10353-017-0507-8 (PMC6223732; doi:10.1007/s10353-017-0507-8)

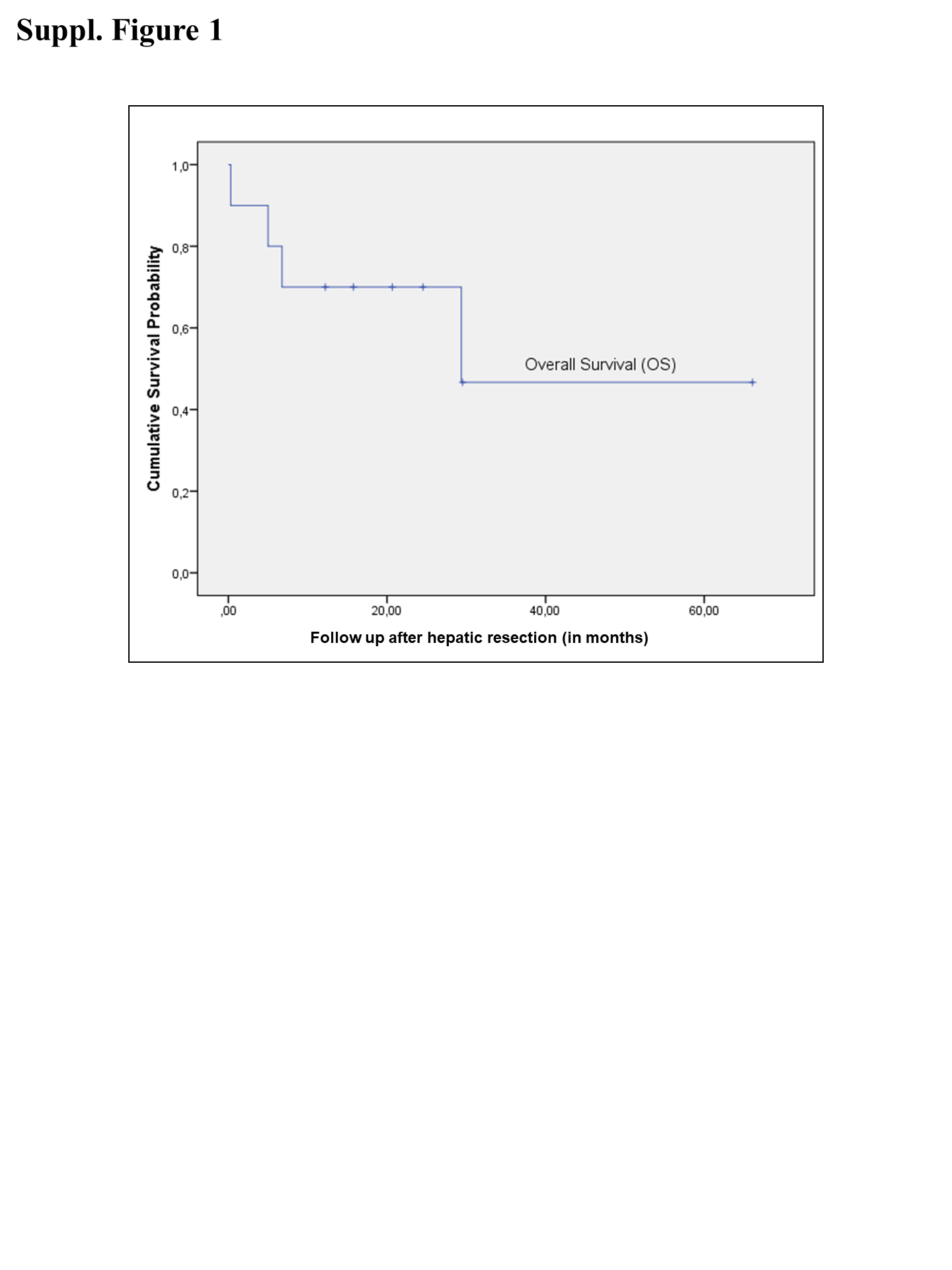

Supplement: Supplementary file 2 — Supplementary Fig. 1 Kaplan–Meier survival curve representing the follow-up and cumulative survival probability after segment 4 partially preserving hilar en bloc resection for hilar cholangiocarcinoma [file 10353_2017_507_MOESM2_ESM.tif]
